# Rebound regrowth phenomenon in patients with pediatric low-grade gliomas treated with MAPK inhibitors – a systematic review

**DOI:** 10.3389/or.2026.1815932

**Published:** 2026-04-29

**Authors:** Malgorzata Styczewska, Weronika Lyzinska, Malgorzata A. Krawczyk, Ewa Bien

**Affiliations:** 1 Department of Pediatrics, Hematology, and Oncology, Medical University of Gdansk, Gdansk, Poland; 2 Department of Pediatrics, Hematology, Oncology, and Immunology, University Clinical Center in Gdansk, Gdansk, Poland

**Keywords:** dabrafenib, low-grade glioma, MAPK inhibitors, rebound regrowth, selumetinib, tovorafenib, trametinib, vemurafenib

## Abstract

**Introduction:**

Mitogen-activated protein kinase pathway inhibitors (MAPKi) are an important therapeutic option for patients with unresectable, symptomatic or progressive pediatric low-grade gliomas (pLGGs). Despite high disease control rates during treatment, tumor regrowth following MAPKi discontinuation has been reported. This phenomenon, termed rebound regrowth (RR), appears biologically and clinically distinct from classic off-therapy progression or acquired resistance to MAPKi. However, the incidence, timing, and predictors of RR remain poorly defined. This systematic review synthesizes evidence on RR following MAPKi discontinuation in pLGG.

**Methods:**

A systematic literature review was conducted according to PRISMA 2020 guidelines. Eligible studies included clinical trials, cohort studies, and case reports/series, involving patients aged 0–25 years with NF1-associated or sporadic pLGG, who experienced RR after MAPKi dose reduction or discontinuation. RR was defined as radiological progression within 6 months after treatment cessation. Cohort-level and individual patient-level data were extracted.

**Results:**

Twenty publications met inclusion criteria. Among ten studies involving ≥10 patients, RR was documented in at least 23 of 131 evaluable individuals. Additionally, individual-level data of 21 patients were analyzed. Most RR events occurred early after MAPKi cessation, were detected radiologically and clinically asymptomatic. RR was reported most frequently in tumors harboring the *BRAF* ^*V600E*^ variant. Most patients responded to MAPKi rechallenge, suggesting preserved drug sensitivity.

**Conclusion:**

RR is a reproducible and clinically relevant phenomenon following MAPKi discontinuation in pLGG. Standardized definitions, structured post-discontinuation surveillance, and prospective evaluation of treatment duration and dose-tapering strategies are needed to optimize MAPKi discontinuation and long-term disease management in patients with pLGG.

## 1 Introduction

Pediatric low-grade gliomas (pLGG), defined as World Health Organization (WHO) grade I and II glial, neuronal, or glioneuronal tumors, are the most common brain tumors in children (1). Circumscribed pLGGs localized in surgically accessible sites are usually treated with resection only, and when excised completely, are associated with favorable long-term outcomes. Conversely, pLGGs that are diffuse and/or located in deep or midline brain structures are often inoperable and pose a significant therapeutic challenge. Although patients with unresectable pLGGs still have excellent overall survival (OS) rates, many of them experience multiple tumor progressions during their lifetime and require systemic treatments to stop pLGG growth and reduce morbidity (2, 3).

In the majority of patients with symptomatic or progressive inoperable pLGGs the standard first-line treatment is chemotherapy (CHT), with carboplatin/vincristine (C/V) regimen and vinblastine (VBL) monotherapy being the most widely used (4, 5). Also, the TPCV scheme (thioguanine, procarbazine, lomustine, and vincristine) has been proposed (6). However, CHT is associated with limited efficacy, with 5-year progression-free survival (PFS) rates ranging from 40% to 45% in sporadic tumors to 70%–85% in neurofibromatosis type 1 (NF1)-associated pLGGs (4, 6). Therefore, many patients require multiple treatment lines to achieve disease control. Radiotherapy (RTX) is used infrequently, mainly as a salvage treatment after several ineffective systemic therapies, as it is associated with significant risk of cognitive impairment, hormonal dysfunction, vasculopathy, malignant tumor transformation, and secondary malignant neoplasms (7–10).

Preclinical and translational studies conducted across recent decades revealed that vast majority of pLGGs harbor molecular alterations activating the RAS/mitogen-activated protein kinase (MAPK) pathway. They include most commonly *BRAF* gene fusions (mainly *KIAA1549–BRAF*), the *BRAF* ^*V600E*^ activating variant, germline NF1 pathogenic variants, and *FGFR1* gene alterations (11–13). Defining the molecular landscape of pLGGs provided rationale for clinical studies evaluating the safety and efficacy of MAPK pathway inhibitors (MAPKi) in patients with pLGGs refractory to conventional therapies. Several phase I and II clinical trials confirmed the efficacy of MAPKi–mainly the drugs targeting BRAF and MAP2K1 (MEK) kinases–in refractory/relapsed pLGGs with *BRAF* alterations and in patients with NF1 (14–17). More recent studies aim to assess the response to MAPKi in the first line of systemic treatment, in comparison to CHT (18). In pLGGs driven by the *BRAF* ^*V600E*^ pathogenic variant, historically associated with a worse response to CHT and unfavorable prognosis (19), the combination of dabrafenib and trametinib led to superior outcomes compared to standard CHT and is currently recommended as the first-line treatment of choice (18, 20). Further clinical trials are ongoing (21). Apart from high disease control rates, treatment with MAPKi offers several other benefits to patients with inoperable pLGGs, including: convenient oral administration, reduced number of hospital stays, and a favorable adverse effects profile as compared to CHT (22).

Despite significant and often sustained responses to MAPKi, a phenomenon of dynamic tumor regrowth following drug discontinuation has been observed (22–24). As its pathophysiology and clinical features seem to differ from the tumor resistance occurring during treatment as well as from the true tumor progression post therapy, the term “rebound regrowth (RR)” was proposed by O’Hare et al (25). This consensus study defined RR as a pLGG regrowth (≥25% per RAPNO criteria (26)) usually within 3 months of cessation of MAPKi therapy (25). The range of 3–6 months was assessed as equivocal, and regrowth occurring ≥6 months after treatment cessation was defined as not being RR. The phenomenon of RR raises doubts about the optimal duration of the treatment with MAPKi. Also, it is still unknown whether slow tapering of the MAPKi dose when drug cessation is planned, or any other strategies, such as combining MAPKi with CHT, prevent RR in patients with pLGG.

Thus, this systematic review aimed to assess the incidence, timing, and predictors of RR after therapy discontinuation or dose reduction in children with pLGG treated with MAPKi, to provide base for further research aimed at optimizing therapeutic outcomes.

## 2 Methods

**FIGURE 1:**
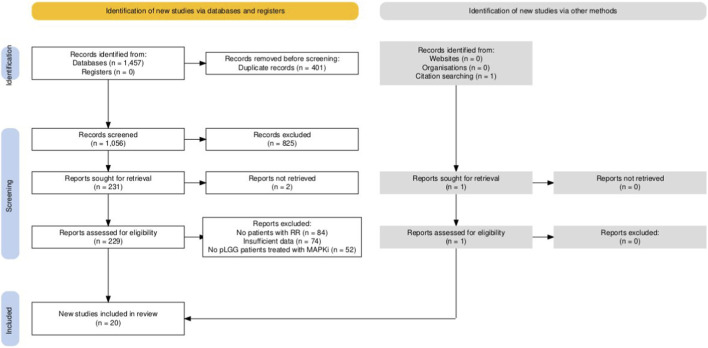
PRISMA flowchart of the systematic review process.

This systematic review was registered in the PROSPERO database (ID: CRD420251142157) and conducted according to the Preferred Reporting Items for Systematic Reviews and Meta-Analyses (PRISMA) 2020 guidelines (Figure 1). PICOS framework of the study is presented in Table 1. Inclusion criteria were studies (clinical trials, prospective/retrospective cohort studies, registries, case series, case reports), reporting patients aged 0–25 years with NF1-associated or sporadic pLGGs, who experienced RR after dose reduction or discontinuation of MAPKi. Dose reduction and treatment discontinuation were defined as a single exposure, as both reflect a spectrum of reduced MAPK pathway inhibition. This approach also allows for a larger sample size to analyze this rare phenomenon. Exclusion criteria were concomitant systemic treatment with CHT, immunotherapy or targeted agents other than MAPKi. Conference abstracts without full texts were included if contained sufficient clinical information. Ongoing clinical trials with no published results were not included in the analysis, but evaluated in the discussion part of the manuscript. pLGGs were defined as WHO grade I–II glial, neuronal, or glioneuronal tumors. Rebound regrowth was defined as radiological tumor progression occurring up to 6 months after the treatment with MAPKi discontinuation (25). Further progression stated in patients who discontinued treatment due to progression on MAPKi therapy was not considered RR. As radiological criteria used to assess the response to treatment in pLGG substantially evolved during recent years, we decided to include also studies using criteria other than RAPNO-LGG (e.g., RANO-HGG, RANO-LGG or volumetric assessments). There was no restriction on language or year of publication.

**TABLE 1:** PICOS framework of the study.

PICOS framework	
Population	Children and adolescents (0–25 years) with NF1-associated or sporadic pediatric low-grade gliomas (pLGG) (WHO grade 1–2 glial, neuronal, and glioneuronal tumors), treated with MAPK pathway inhibitors (MAPKi), who experienced rebound regrowth (RR) within ≤6 months after dose reduction or discontinuation
Intervention/exposure	Treatment with MAPKi, including dose reduction or discontinuation scenarios
Comparison	No direct comparison group However, implicit comparison has been conducted between patients with vs. without RR (exploratory comparisons for predictors)
Outcome	○ Incidence of rebound regrowth (RR) ○ Time from last dose (or full dose in reduction cases) to RR ○ Clinical deterioration at RR (new/worsened symptoms) ○ Management strategies for RR ○ Response to MAPKi re-challenge ○ Predictors of RR ○ Long-term outcomes after RR
Study design	Systematic review of clinical trials, prospective/retrospective cohort studies, registries, case series, case reports, and meeting abstracts. Conducted per PRISMA 2020 guidelines, registered in PROSPERO (ID: CRD420251142157)

Publications listed in the PubMed/MEDLINE, Embase, Cochrane Central Register of Controlled Trials (CENTRAL), Scopus, Web of Science, clinicaltrials.gov and International Clinical Trials Registry Platform (ICTRP) databases up to 06.09.2025 were searched. The screening (database search, article retrieval, and data analysis) was performed by two independent reviewers (M.S., W.L.). Duplicate publications were removed using an automatic tool (Rayyan (27)), and manually. The search was performed using both subject headings (MeSH and Emtree terms in MEDLINE and Embase, respectively) and free-text keywords. Search terms and the full search protocol are provided in the Supplementary File 1. No additional search filters were applied. The reference lists in all publications were searched for additional studies.

Retrieved information included both data from entire study cohorts (for studies involving more than 10 patients with pLGG treated with MAPKi) and, where available, individual-level data of patients who experienced RR. Assessed outcomes for entire cohorts were study design and location, inclusion and exclusion criteria, number of patients who received and discontinued MAPKi treatment, details on the MAPKi treatment (name of the drug, dose, treatment duration, setting–upfront vs. refractory/relapsed), reasons for MAPKi discontinuation, definition and incidence of RR, time from the last MAPKi dose (full, in case of dose reduction) to RR, management of RR (including response to MAPKi rechallenge), predictors of RR, and follow-up. Additionally, collected individual-level data included patient age and sex, tumor location, WHO diagnosis, molecular tumor drivers, therapies preceding MAPKi treatment, and detailed information on the disease course, including RR management.

A structured risk-of-bias assessment was performed based on Joanna Briggs Institute (JBI) critical appraisal principles (28), tailored to the heterogeneity of included studies (clinical trials, retrospective cohorts, and case series). Domains assessed included selection bias, exposure measurement, outcome measurement (including definition of RR), follow-up completeness and reporting bias. Each item was rated as “yes,” “no,” “unclear,” or “not applicable,” with “yes” indicating lower risk of bias, and domain-level and overall judgments (low, moderate, high) were assigned accordingly. The assessment was performed independently by two reviewers (M.S., W.L.), with discrepancies resolved through discussion. The results of the risk-of-bias assessment were used to inform interpretation of the evidence.

## 3 Results

The systematic literature search identified 20 studies on patients with pLGG treated with MAPKi, reporting at least one patient with RR (14, 15, 17, 22–24, 29–42). Among them, there were ten studies involving more than 10 patients. Additionally, data of 21 individual patients from studies, case-reports and case-series were retrieved (Figure 1; Tables 2, 3, Supplementary File 2). Several patients are included both in cohort-based and individual patient-based analyses (17, 31, 32, 34, 35, 38).

**TABLE 2:** Data of included studies involving ≥10 patients with pLGG treated with MAPKi.

Study	Population	Treatment	Radiological response assessment criteria	Rebound regrowth (RR)	Outcomes
(14) Phase I	Inclusion criteria: age ≥3 and ≤21 years, progressive/recurrent pLGG, ≥1 prior treatment	Drug: Selumetinib	T2/FLAIR volumetric (initially also T1 with contrast enhancement)	Time from MAPKi discontinuation to RR: N/A (50% within 3 months, 90% within 7 months)	N/A
	Exclusion criteria: prior MEKi or BRAFi therapy	Duration: 13 cycles (1–26 cycles) – approx. 12 months (1–24 months)			
	NF1 status: mixed				
	38 treated → 38 stopped treatment (including 7 due to PD) → 5 experienced RR (4 more patients progressed 3–7 months following MAPKi discontinuation – no individual data)	Reasons for discontinuation: PD (7), planned (14), toxicity/patient’s decision (17)		Risk factors: N/A	
(36) Retrospective	Inclusion criteria: age <18 years, nonresectable, symptomatic, progressive pLGG, documented *BRAF* alteration	Drug: vemurafenib, dabrafenib, trametinib, tovorafenib (monotherapy and combinations)	N/A	Time from MAPKi discontinuation to RR: mean: 4.1 months (N/A if all patients met criteria of RR)	PR to rechallenge with MAPKi in all patients
	Exclusion criteria: N/A	Duration: for patients who discontinued MAPKi: mean: 23 months (range 12–36 months)			
	NF1 status: N/A			Risk factors: N/A	
	19 treated → 8 stopped treatment (6: *BRAF V600E*-positive; 2 with *KIAA1549::BRAF* fusion) → 6 experienced RR	Reasons for discontinuation: planned (8)			
(15) Phase I/II	Inclusion criteria: age 1 to <18 years, *BRAFV600E*-positive, refractory/relapsed pLGG, ≥1 prior treatment	Drug: dabrafenib	RANO	Time from MAPKi discontinuation to RR: N/A	N/A; follow-up: minimum: 26.2 months
	Exclusion criteria: in part 2 of the study – prior MEKi or RAFi therapy	Duration: approx. 25 months (<1–43 months)			
	NF1 status: N/A			Risk factors: N/A	
	32 treated → 17 stopped treatment (including 4 due to PD) → 3 experienced RR	Reasons for discontinuation: investigator’s discretion (11), progression (4) toxicity (2)			
(17) Phase I	Inclusion criteria: age <18 years, *BRAFV600E*-positive primary brain tumor, ≥1 prior treatment regimen (including RTX)	Drug: vemurafenib	Modified RANO-HGG (assessment in T2/FLAIR, including cystic components)	Time from MAPKi discontinuation to RR: median: 2.2 months; range: 1.5–3.2 months	N/A; follow-up: ≥12 months following discontinuation
	Exclusion criteria: prior BRAFi therapy, suspicion of keratoacanthoma or cutaneous squamous cell carcinoma, NF1 and other RASopathies	Duration: 23 courses (4–64 courses) approx. 22 months (4–59 months)			
	NF1 status: negative			Risk factors: N/A	
	19 treated → 15 stopped (including 1 due to PD) → 3 experienced RR	Reasons for discontinuation: toxicity or patient’s decision (11), planned (3) progression (1)			
(22) Retrospective	Inclusion criteria: age <25 years, *BRAF V600E*–positive gliomas treated with BRAFi monotherapy outside clinical trials; follow-up ≥ 6 months	Drug: vemurafenib, dabrafenib	Modified RANO-LGG	Time from MAPKi discontinuation to RR: median: 2.3 months; range 0.3–20.8 months (not all patients met criteria of RR)	9/13 patients rechallenged with BRAFi, in monotherapy (n = 8) or in combination with MEKi (n = 1); OR in all patients except one, who required dose reduction
	Exclusion criteria: N/A	Duration: N/A			
	NF1 status: N/A			Risk factors: N/A	
	56 treated → 17 stopped → RR: N/A (insufficient individual data); median time to RR among 13 patients: 2.3 months	Reasons for discontinuation: patient/physician decision (14), toxicity (3)			
(38) Retrospective	Inclusion criteria: pLGG, *KIAA1549-BRAF-*positive or *BRAF V600E-*positive	Drug: trametinib, dabrafenib	RAPNO	Time from MAPKi discontinuation to RR: 3 months; 2 months (single patient with 2 RR events)	MAPKi rechallenge → CR (both events); follow-up: 25.6 months (range: 4–54)
	Exclusion criteria: other abnormalities of the MAPK pathway, NF1	Duration: trametinib: 12 months (range: 6–21) dabrafenib: 15 months (range: 4–27			
	NF1 status: negative			Risk factors: N/A	
	23 treated → 10 stopped (including 3 due to PD) → 1 experienced RR (additional 1 with drug re-introduction, but insufficient data)	Reasons for discontinuation: progression (3), poor efficacy not fulfilling progression criteria (1), unknown (6)			
(30) Phase II	Inclusion criteria: patients in FIREFLY-1 (with relapsed/progressive pLGG with activating *BRAF* alteration), treated for ≥26 cycles (∼24 months), who entered a drug holiday period	Drug: tovorafenib	RANO-LGG	Time from MAPKi discontinuation to RR: 1 month (single patient)	Rechallenge (on treatment for 4 months); no information on response
	Exclusion criteria: activating molecular alterations other than *BRAF*; NF1	Duration: ∼24 months			
	NF1 status: negative			Risk factors: N/A (the patient had BRAFV600E-positive tumor)	
	77 treated → 26 stopped (24 evaluable) → 1 experienced RR	Reasons for discontinuation: treatment protocol (26 – all)			
(35) Retrospective	Inclusion criteria: unresectable *BRAFV600E*-positive brain pLGG, RTX naïve	Drug: N/A	RAPNO	Time from MAPKi discontinuation to RR: 4 days (single patient)	MAPKi rechallenge → PR; follow-up: 4.1 years (range 2.5–8.7 years)
	Exclusion criteria: N/A	Duration: N/A			
	NF1 status: N/A			Risk factors: N/A	
	12 treated → unknown number stopped → 1 experienced RR	Reasons for discontinuation: N/A			
(31) Retrospective	Inclusion criteria: pLGG patients treated with trametinib *off label*	Drug: trametinib	RAPNO (with modifications), volumetric	Time from MAPKi discontinuation to RR: median: 3 months; range: 2–4 months	One patient: rechallenge with trametinib → SD; one patient: VBL, one patient: no treatment (spontaneous stabilization); follow-up: N/A – available only for a subgroup of 9 patients who discontinued trametinib: median of 7 months (range 1–33)
	Exclusion criteria: N/A	Duration: 12.5 months (2–27 months)			
	NF1 status: mixed			Risk factors: N/A	
	18 treated → 11 stopped (one with no follow-up) → 3 experienced RR (additional 1 patient with tumor regrowth assessed as SD on imaging, but requiring treatment)	Reasons for discontinuation: N/A			
(29) Retrospective	Inclusion criteria: pLGGs treated with targeted therapy *off-label*	Drug: trametinib, dabrafenib, vemurafenib	RAPNO, RANO, volumetric (used to calculate regrowth)	Time from MAPKi discontinuation to RR: median time to largest postcessation volume: in 5 *BRAFV600E-*positive patients after BRAFi treatment: 2.86 months; in 4 *BRAF* fusion/duplication patients after MEKi treatment: 2.38 months	5 *BRAFV600E-*positive patients after BRAFi treatment: mean largest postcessation volume increase: 13.49% 4 *BRAF* fusion/duplication patients after MEKi treatment: mean largest post-cessation volume increase: 71.86%; two of them discontinued MAPKi treatment due to PD 4 of 9 patients received further treatments (tovorafenib: 2; V/C: 1; dabrafenib rechallenge: 1) follow-up: 5 *BRAFV600E-*positive patients after BRAFi treatment: median 0.90 years postcessation (range 0.21–3.24 years) 4 *BRAF* fusion/duplication patients after MEKi treatment: median 0.43 years postcessation (range 0.07–0.99 years)
	Exclusion criteria: treatment within clinical trial	Duration: dabrafenib: approx. 14 months (6–26 months); trametinib: approx. 5 months (0.1–6 months); vemurafenib: approx. 11 months (8–13 months)			
	NF1 status: N/A			Risk factors: more significant tumor volume increase in patients with *BRAF* fusion/duplication after cessation of MEKi compared to patients with *BRAFV600E* after cessation of BRAFi	
	29 treated → 18 stopped (9 evaluable - trametinib: 4, including 2 due to PD; dabrafenib: 3; vemurafenib: 2) → unknown number experienced RR (insufficient data)	Reasons for discontinuation: toxicity (6), progression (5), scheduled discontinuation (7)			

**TABLE 3:** Individual-level patient data.

All patients	n = 21 (100%)
With one RR	n = 19 (90.5%)
With two RRs	n = 2 (9.5%)
All RR events	n = 23 (100%)
Sex	
Female	n = 8 (38.1%)
Male	n = 4 (19%)
N/A	n = 9 (42.9%)
Median age at diagnosis [years]	5 (range: 0.5–16; IQR: 2.33–14)
Tumor site	
Brainstem	n = 6 (28.6%)
Hypothalamic-chiasmatic area	n = 3 (14.3%)
Brainstem + spinal cord	n = 2 (9.5%)
Temporal lobe	n = 2 (9.5%)
Third ventricle + thalamus + midbrain	n = 1 (4.8%)
Cerebellum + leptomeningeal dissemination	n = 1 (4.8%)
N/A	n = 6 (28.6%)
NF1 status	
Negative	n = 5 (23.8%)
Positive	n = 1 (4.8%)
N/A	n = 15 (71.4%)
Histopathological diagnosis	
Ganglioglioma	n = 9 (42.9%)
Pilocytic astrocytoma	n = 7 (33.3%)
pLGG NOS	n = 2 (9.5%)
Pleomorphic xanthoastrocytoma	n = 2 (9.5%)
Diffuse astrocytoma	n = 1 (4.8%)
Molecular alteration	
*BRAFV600E*	n = 15 (71.4%)
*BRAFV600E* + *CDKN2A* deletion^*^	n = 2 (9.5%)
*KIAA1549:BRAF* fusion	n = 2 (9.5%)
Germline *NF1* (clinical)	n = 1 (4.8%)
*FGFR1* N546K	n = 1 (4.8%)
MAPKi treatment setting	
Refractory/relapsed	n = 17 (81%)
Upfront	n = 2 (9.5%)
N/A	n = 2 (9.5%)
Drug	
Vemurafenib	n = 12 (57.1%)
Dabrafenib	n = 4 (19%)
Trametinib	n = 3 (14.3%)
Selumetinib	n = 1 (4.8%)
Dabrafenib + trametinib	n = 1 (4.8%)
Median MAPKi treatment duration [months]	23 (range: 0.25–72; IQR 13–24)
Reason for MAPKi discontinuation (n = 23; all RR events)	
Planned end of therapy	n = 10 (43.5%)
Toxicity	n = 3 (13%)
Patient/parent decision	n = 2 (8.7%)
Hypersensitivity reaction	n = 1 (4.3%)
Neurological deterioration	n = 1 (4.3%)
N/A	n = 6 (26.1%)
Median time from MAPKi discontinuation to RR	2 months (range: 6 days–6 months IQR 1.5–3 months)
First treatment following RR (n = 23; all RR events)	
Dabrafenib	n = 7 (30.4%)
Dabrafenib + trametinib	n = 5 (21.7%)
Vemurafenib	n = 3 (13%)
Selumetinib	n = 1 (4.3%)
Trametinib	n = 1 (4.3%)
Vinblastine	n = 1 (4.3%)
No treatment	n = 1 (4.3%)
N/A	n = 4 (17.4%)
Response to the first treatment of RR (n = 23; all RR events)	
CR	n = 2 (8.7%)
PR	n = 11 (47.8%)
SD	n = 1 (4.3%)
PD	n = 1 (4.3%)
N/A	n = 8 (34.8%)
Outcome	
AWD	n = 16 (76.2%)
NED	n = 1 (4.8%)
N/A	n = 4 (19%)
Median follow-up following last RR [months]	13 (range: 1–78; IQR: 8.25–30)

* one homozygous deletion, one unknown.

AWD, alive with disease; IQR, interquartile range; MAPKi, MAPK, pathway inhibitor; N/A = not available; NED, no evidence of disease; RR, rebound regrowth; TPCV, thioguanine, procarbazine, lomustine, and vincristine.

### 3.1 Clinical trials and observational studies involving ≥10 patients

#### 3.1.1 Study selection and general characteristics

All identified studies were published between 2017 and 2024. Four publications represented international collaborations. There were six retrospective studies and four prospective clinical trials–two of the phase I, one of the phase I/IIa, and one of the phase II. None of the included studies were primarily designed to investigate RR as a predefined endpoint. Instead, data on RR were reported as a secondary observation or *post hoc* finding during follow-up after MAPKi treatment discontinuation. Overall, the risk of bias across studies was high (Supplementary Files 3, 4). The main sources of bias included retrospective design, lack of predefined RR criteria, heterogeneous outcome definitions, and incomplete follow-up data.

#### 3.1.2 Study Populations

Overall, the ten studies reported outcomes for 323 patients with pLGGs treated with MAPKi. Age eligibility criteria varied slightly between studies but generally encompassed children, adolescents, and young adults up to 18–25 years of age. Five out of ten studies reported the NF1 status of the patients–there were only patients without NF1 in three studies, and patients with and without NF1 in two. In seven studies only patients with documented BRAF alterations were recruited, including four studies designed specifically for patients with BRAF^V600E^-positive tumors. Four studies included exclusively patients with refractory/relapsed pLGG, in the remaining six there was also a minority of patients treated in the upfront setting.

#### 3.1.3 MAPK inhibitor treatment and its discontinuation

The studies evaluated patients treated with vemurafenib (1), dabrafenib (1), selumetinib (1), trametinib (1), tovorafenib (1), or several various MAPKi, administered as monotherapy or in combination (4). In one study, there was no information which drug was administered. The number of patients treated with MAPKi in each study ranged from 12 to 77 (median: 26); among them a median of 17 patients (range: 8–38) discontinued therapy. The most common reasons for MAPKi discontinuation included completion of planned therapy protocol, toxicity, and patient/physician preference. Importantly, a subset of discontinuations occurred due to disease progression on therapy, and these patients were excluded from RR analyses in accordance with the predefined criteria. The radiological criteria used to evaluate the response to MAPKi treatment were heterogeneous and included RAPNO-LGG, RANO-LGG and RANO-HGG criteria, volumetric assessment and combinations thereof.

#### 3.1.4 Rebound regrowth phenomenon

RR was reported in at least 23 of 131 (17.6%) evaluable patients who discontinued MAPKi therapy across the ten studies. The exact RR incidence rate in individual studies could not be reliably calculated due to heterogeneity in reporting and incomplete data. However, RR consistently affected a substantial proportion of patients discontinuing MAPKi therapy. There were no reports on RR phenomenon following dose reduction of MAPKi without complete drug discontinuation. Importantly, several studies reported that RR could be effectively managed by re-initiation of MAPKi, suggesting preserved treatment sensitivity of pLGG. Follow-up duration after RR was limited and inconsistently reported, precluding definitive conclusions regarding long-term outcomes.

### 3.2 Individual-level patient data

Among 21 identified patients with available individual-level data, two experienced RR twice, resulting in a total of 23 RR events (Table 3, Supplementary File 2). The median age at pLGG diagnosis was 5 years (range: 0.5–16; IQR: 2.33–14). Among 12 children with known sex, there was a female predominance (8 girls, 4 boys). Tumors were most frequently located in the brainstem (n = 6, 28.6%), followed by the hypothalamic–chiasmatic region (n = 3, 14.3%), the brainstem and spinal cord (n = 2, 9.5%), and the temporal lobe (n = 2, 9.5%). Histopathological diagnoses included ganglioglioma (n = 9, 42.9%), one of which exhibited anaplastic features; pilocytic astrocytoma (n = 7, 33.3%), pleomorphic xanthoastrocytoma (n = 2, 9.5%) and diffuse astrocytoma (n = 1, 4.8%). In two patients (9.5%), the specific LGG subtype was not determined. Twenty tumors (95.2%) underwent molecular testing, revealing *BRAF* ^*V600E*^ pathogenic variant in 17 cases (81%), including two patients with concomitant *CDKN2A* deletion (9.5%), a KIAA1549:BRAF fusion in 2 patients (9.5%), and an *FGFR1* N546K mutation in one child (4.8%). Only one patient (4.8%) was NF1-positive; five (23.8%) had no NF1, and NF1 status was not provided in the remaining cases.

In 17 patients (81%), MAPKi were administered in the refractory or relapsed setting, while two children (9.5%) received targeted therapy as the first line therapy. In the remaining two cases (9.5%), the setting of MAPKi initiation was not specified. The agents used included vemurafenib (n = 12, 57.1%), dabrafenib (n = 4, 19.0%), trametinib (n = 3, 14.3%), selumetinib (n = 1, 4.8%), and dabrafenib/trametinib combination (n = 1, 4.8%). The median duration of MAPKi treatment was 23 months (range: 0.25–72; IQR 13–24). All patients discontinued MAPKi without dose tapering. Treatment cessation was planned in 10 of the 23 situations in which RR was observed (43.5%), whereas toxicity prompted discontinuation in 3 cases (13%). Other reasons included patient/parent decision (n = 2, 8.7%), hypersensitivity reaction (n = 1, 4.3%), and neurological deterioration (n = 1, 4.3%). In six cases (26.1%), the reason for discontinuation was not reported. The median time from MAPKi cessation to RR was 2 months (range: 6 days–6 months; IQR 1.5–3 months). Only a minority of RR events (n = 8; 34.8%) were reported to be clinically symptomatic, while the majority were detected radiologically.

The subsequent treatment line following RR included dabrafenib (n = 7, 30.4%), dabrafenib/trametinib combination (n = 5, 21.7%), followed by vemurafenib (n = 3, 13%), selumetinib (n = 1, 4.3%), trametinib (n = 1, 4.3%) and vinblastine (n = 1, 4.3%). In 11 cases, the patients received the same MAPKi as before RR, whereas in six cases different agents were used (dabrafenib +/- trametinib in patients previously treated with vemurafenib or selumetinib). Altogether, in almost all situations (13 out of 15 with available outcome data, 86.7%), the patients responded to MAPKi reintroduction following RR. Among the patients who did not respond to MAPKi reintroduction, one received dabrafenib (following dabrafenib/trametinib treatment before RR) and was later switched to the TPCV + bevacizumab regimen, and one had SD on re-introduced trametinib.

At the time of publication, 16 children (76.2%) were alive with disease (AWD), one patient (4.8%) had no evidence of disease (NED), and outcome data were unavailable for four children (19%). The median follow-up time after RR (available for 16 RR events) was 13 months (range: 1–78 months; IQR: 8.25–30 months).

## 4 Discussion

The introduction of MAPKi has substantially transformed the therapeutic landscape for patients with pLGGs. These agents provide high disease control rates with rapid and durable responses (15, 22). However, the optimal treatment duration and discontinuation strategies remain undefined. Moreover, up to 40% of patients treated with MAPKi require unplanned dose reductions or drug cessation due to adverse effects (16, 22, 31). It is unknown how to best manage these clinical situations to avoid rapid tumor progression. In this systematic review, we summarize data from ten studies and 21 individual patients with pLGG who experienced RR following MAPKi discontinuation, confirming that RR is a phenomenon observed across different clinical contexts and various MAPKi agents.

A phenomenon of accelerated tumor growth after cessation of tyrosine kinase inhibitors (TKIs) has been reported in patients treated for other malignancies, providing context for RR in pLGG. Rapid tumor progression following TKIs withdrawal was initially observed in adults with renal cell carcinoma (RCC) treated with sunitinib, including growth of brain metastases during short off-treatment intervals, which were part of standard therapeutic protocols (43–45). Similar phenomena were reported in patients with non-small cell lung cancer after stopping administration of gefitinib or erlotinib and in patients with thyroid carcinoma following cessation of lenvatinib or sorafenib (46–49). Rapid recurrences after TKI discontinuation were observed even in patients with complete response after the initial treatment. In many cases, responses to drug rechallenge were reported (43, 44, 50, 51). A very high recurrence rates following MAPKi treatment discontinuations were reported in melanoma, Erdheim–Chester disease and Langerhans cell histiocytosis (52–55). However, these comparisons (especially with aggressive adult malignancies) should be interpreted with caution, as pLGG exhibit a relatively indolent biology, and therefore the characteristics of RR may differ in this setting.

The biological mechanisms underlying RR in pLGG remain incompletely understood but appear to reflect adaptive responses to chronic MAPK pathway suppression. Sustained inhibition of MAPK signaling can induce compensatory feedback activation of upstream or parallel pathways, leading to MAPK pathway hyperactivation upon drug withdrawal. In particular, ERK-dependent negative feedback normally suppresses signaling through receptor tyrosine kinases (RTKs) and RAS; its attenuation during MAPKi therapy results in RTK upregulation and increased RAS activation, which can rapidly restore downstream MAPK signaling once the inhibition is removed (56). This hypothesis is supported by the results of a study by Kocher et al., which showed that in a preclinical model of *BRAF* ^*V600E*^-mutant pLGG with concurrent *CDKN2A/B* deletion, RR following MAPKi discontinuation was associated with a rapid MAPK pathway reactivation. Additionally, it was suggested that microglia recruitment through cytokine secretion may influence the response to therapy and contribute to RR (57). Emerging data also suggest that MAPK inhibition may induce adaptive cellular states, including increased cellular plasticity and transcriptional reprogramming toward a progenitor-like phenotype, which could facilitate rapid tumor regrowth upon drug withdrawal (58, 59). In clinical setting, the temporal proximity to MAPKi cessation and the accelerated tumor growth kinetics support the hypothesis that RR represents a biologically distinct phenomenon rather than natural disease progression off treatment. Additionally, the almost universally observed response to MAPKi rechallenge suggests that the MAPK pathway hyperactivation in RR is reversible.

The exact incidence of RR in patients with pLGG following MAPKi treatment discontinuation is difficult to assess. In this systematic review, across ten studies encompassing 131 evaluable patients with pLGG who discontinued MAPKi therapy, RR was documented in at least 23 individuals (17.6%). Although precise incidence rates in each study could not be calculated, RR consistently affected a substantial proportion of patients. In the majority of cases, RR occurred early after MAPKi withdrawal–typically within weeks to a few months. Rebound regrowth appeared to occur independent of specific MAPKi used, suggesting a drug class effect. However, as only one RR event was observed among patients who started “drug holidays” following 26 courses of tovorafenib treatment in the FIREFLY-1 trial, it seems that the incidence of RR following pan-RAF inhibitors may be lower compared to earlier generation drugs, although these findings should be interpreted with caution given the relatively short post-discontinuation follow-up (range: 0.6–10.9 months), limiting detection of delayed events (30). This is particularly relevant given the distinct pharmacokinetic properties of pan-RAF inhibitors, suggesting that adaptive MAPK reactivation–and consequently RR–may occur later than with other MAPKi. Therefore, the ≤6-month definitional threshold of RR may underestimate its incidence in this setting, underscoring the need for extended surveillance in future pan-RAF inhibitor cohorts.

Radiological criteria used to assess tumor response in analyzed studies were very heterogenous, including RANO-HGG, RANO-LGG, RAPNO-LGG and their modifications, precluding direct comparisons (26, 60–62). Current recommendations emphasize T2/FLAIR-based sequences (according to the RAPNO-LGG criteria) for evaluation of suspected RR, as the commonly observed rapid increase in contrast enhancement on T1-weighted sequences following MAPKi discontinuation may not necessarily reflect true tumor progression (15, 25). Moreover, as demonstrated by Tsai et al., radiological features consistent with progression may be transient, therefore in asymptomatic patients a watchful waiting strategy may be appropriate (29). Difficulties to distinguish RR from true tumor progression result in increased risk of unnecessary shift to more toxic therapies, including RTX.

Analysis of individual patient-level data provides further insights. All reported RR events occurred after abrupt MAPKi discontinuation without dose tapering, and the median time to RR was short (2 months), with several cases developing RR within weeks or even days. Importantly, approximately one-third of RR events (34.8%) were reported to be clinically symptomatic, whereas most were detected radiologically during routine surveillance. This finding underscores the importance of structured imaging follow-up, particularly during the first 6 months after MAPKi cessation. No RR-related mortality was observed. Most patients responded to MAPKi rechallenge, either with the same agent or an alternative MAPKi-based regimen, indicating preserved treatment sensitivity. These findings suggest that RR, when promptly recognized, is clinically manageable and does not necessarily result in an adverse long-term outcome.

Currently, no validated clinical or molecular biomarkers can reliably predict the risk of RR. *BRAF* ^*V600E*^-positive tumors appear overrepresented among individual patients reported to experience RR (17 out of 21 patients). Similarly, Nobre et al. reported that 13 out of 17 patients with *BRAF* ^*V600E*^-mutated pLGG who discontinued MAPKi treatment experienced tumor progression, with a median time to progression of 2.3 months. In this group, a 1-year PFS for patients who discontinued MAPKi after ≥10 months of treatment was only 26.7% (22). However, the apparent predominance of *BRAF* ^*V600E*^-positive tumors among reported RR cases may also reflect earlier and broader access to MAPKi in this subgroup, compared to patients with other molecular alterations. Interestingly, Tsai et al. reported opposite observations, as considerable tumor regrowth (mean volume increase of 71.86%) was found in 4 patients with *BRAF* fusions after trametinib discontinuation, whereas there were no cases of RR among 5 patients with *BRAF* ^*V600E*^ *-*mutated tumors treated with vemurafenib of dabrafenib. These latter patients experienced transient, mild growth not fulfilling RR criteria (mean volume increase of 13.49%) shortly following treatment cessation, with subsequent spontaneous reduction in tumor volume without further treatment (29). However, this cohort was small and retrospective. Moreover, two of the patients in the trametinib group discontinued MAPKi treatment due to tumor progression on therapy (so the tumor regrowth after therapy cessation should not be interpreted as typical RR). These observations suggest that molecular subtype may influence RR risk, although available data are too limited and heterogeneous to confirm this suggestion.

The optimal duration of MAPKi treatment and the role of gradual dose tapering during treatment discontinuation are still unknown. It has been hypothesized that longer therapy may decrease the risk of RR; however, there is no direct clinical evidence to support this hypothesis. Canadian expert consensus proposed a 36-month duration of MAPKi treatment period for *BRAF* ^*V600E*^-mutated LGG, with a suggestion to consider tapering of the drug dose in case of complete response or minimal residual disease, favorably located tumors (when significant neurological deterioration upon progression is not anticipated) or presence of substantial adverse effects. Proposed tapering pace was 25% every 3 months for MEK inhibitors (MEKi), and 25% every 6 months for BRAF inhibitors (BRAFi) (20). Among literature patients, all experienced RR following rapid MAPKi discontinuation without dose tapering; however, these observations are limited and do not provide definitive evidence that slow dose tapering decreases the risk of RR development. The currently recruiting PNOC037 trial (NCT07110246) may provide new insights by comparing abrupt discontinuation *versus* gradual dose reduction of dabrafenib and trametinib in patients with BRAF^V600^–mutated pLGG, with the primary endpoint of RR and/or clinical progression within 4 months after treatment cessation.

Patients with pLGG who develop acquired resistance to MAPKi (i.e., patients with tumor progression on treatment) may experience further, accelerated tumor growth following therapy discontinuation (29). A similar phenomenon has been reported in patients with melanoma (63). Therefore, the decision regarding treatment withdrawal in patients with tumor progression during MAPKi therapy should be very cautious, especially in the presence of neurological symptoms or a high risk of morbidity if further progression occurs. When feasible, initiating of an alternative treatment immediately after MAPKi cessation may help avoid prolonged off-treatment intervals.

## 5 Conclusion

Rebound regrowth following MAPKi discontinuation is a reproducible and clinically significant phenomenon in pLGGs. Physicians treating patients with pLGGs should be aware of the risks associated with possible occurrence of RR following MAPKi cessation. The risk of RR should be considered during multidisciplinary team discussions and discussed with the patients and their parents/caregivers while obtaining informed consent for MAPKi treatment. Patients who discontinued MAPKi should remain under close clinical and radiological surveillance, especially during first 6 months following the end of treatment.

Further research on RR phenomenon should integrate biological correlates, such as molecular tumor profiling, which may help elucidate the mechanisms driving RR and identify patients at highest risks. Prospective clinical trials for patients with pLGG should aim to analyze RR phenomenon as a predefined outcome with standardized definitions, uniform imaging criteria, and structured post-discontinuation follow-up. A *post hoc* analysis of already published trials to assess the risk factors associated with RR should be considered. This, in the future, may lead to development of evidence-based MAPKi discontinuation guidelines for patients with pLGG.

## 6 Limitations of the study

The RR was not the primary endpoint of analyzed clinical studies; therefore, they lacked detailed information on the disease course and follow-up of patients exhibiting this phenomenon. Heterogeneity of definitions of RR and radiological response assessment criteria (RANO-HGG, RANO-LGG, RAPNO-LGG, volumetric) precluded reliable comparison of outcomes in the different study cohorts. The high risk of bias across included studies substantially limits the certainty of conclusions.

## Data Availability

Publicly available datasets were analyzed in this study. This data can be found here: PubMed/MEDLINE, Embase, Cochrane Central Register of Controlled Trials (CENTRAL), Scopus, and Web of Science databases.
