# Supplementary material for: Rebound regrowth phenomenon in patients with pediatric low-grade gliomas treated with MAPK inhibitors – a systematic review
Source: Oncol Rev. 2026 Apr 29;20:1815932. doi: 10.3389/or.2026.1815932 (PMC13168116; doi:10.3389/or.2026.1815932)
Supplement: Supplementary file 1 [file Supplementaryfile1.docx]

1. PUBMed/MEDLINE (title/abstract + MeSH terms)

| pediatric | AND | glioma | AND | BRAF | AND | rebound |
| --- | --- | --- | --- | --- | --- | --- |
| paediatric |  | astrocytoma |  | MAPK |  | re-bound |
| childhood |  | glioneuronal |  | MEK |  | „tumor flare” |
| children |  | ganglioglioma |  | MAP2K1 |  | „tumour flare” |
| infant |  | neuroepithelial |  | Targeted |  | „rapid progression” |
| adolescent |  | xanthoastrocytoma |  | Raf |  | regrowth |
|  |  | neurocytoma |  | Selumetinib |  | cessation |
| Child [MeSH] |  |  |  | Trametinib |  | withdraw* |
| Adolescent [MeSH] |  | Glioma [MeSH] |  | Binimetinib |  | discontinuat* |
| Infant [MeSH] |  |  |  | Cobimetinib |  | stop |
|  |  |  |  | mirdametinib |  | taper |
|  |  |  |  | refametinib |  | deescalat* |
|  |  |  |  | Dabrafenib |  |  |
|  |  |  |  | vemurafenib |  |  |
|  |  |  |  | Encorafenib |  |  |
|  |  |  |  | tovorafenib |  |  |
|  |  |  |  | DAY101 |  |  |
|  |  |  |  |  |  |  |
|  |  |  |  | Mitogen-Activated Protein Kinase Kinases [MeSH] |  |  |

+ additional search: first 3 columns only and filters „trial” and “clinial trial” – to search manually information in trials

2. EMBASE

| pediatric | AND | glioma | AND | BRAF | AND | rebound |
| --- | --- | --- | --- | --- | --- | --- |
| paediatric |  | astrocytoma |  | MAPK |  | re-bound |
| childhood |  | glioneuronal |  | MEK |  | „tumor flare” |
| children |  | ganglioglioma |  | MAP2K1 |  | „tumour flare” |
| infant |  | neuroepithelial |  | Targeted |  | „rapid progression” |
| adolescent |  | xanthoastrocytoma |  | Raf |  | regrowth |
|  |  | neurocytoma |  | Selumetinib |  | cessation |
| Child [EmTree] |  |  |  | Trametinib |  | withdraw* |
| Adolescent [EmTree] |  | Glioma [EmTree] |  | Binimetinib |  | discontinuat* |
|  |  |  |  | Cobimetinib |  | stop |
|  |  |  |  | mirdametinib |  | taper |
|  |  |  |  | refametinib |  | deescalat* |
|  |  |  |  | Dabrafenib |  |  |
|  |  |  |  | vemurafenib |  |  |
|  |  |  |  | Encorafenib |  |  |
|  |  |  |  | tovorafenib |  |  |
|  |  |  |  | DAY101 |  |  |
|  |  |  |  |  |  |  |
|  |  |  |  | protein serine threonine kinase [EmTree] |  |  |
|  |  |  |  | B Raf kinase inhibitor [EmTree] |  |  |
|  |  |  |  | mitogen activated protein kinase inhibitor [EmTree] |  |  |
|  |  |  |  | mitogen activated protein kinase kinase inhibitor [EmTree] |  |  |
|  |  |  |  | Tovorafenib [EmTree] |  |  |

+ additional search: first 3 columns only and filters „trial” and “clinial trial” – to search manually information in trials

3. COCHRANE, Web of Science, Scopus

| pediatric | AND | glioma | AND | BRAF | AND | rebound |
| --- | --- | --- | --- | --- | --- | --- |
| paediatric |  | astrocytoma |  | MAPK |  | re-bound |
| childhood |  | glioneuronal |  | MEK |  | „tumor flare” |
| children |  | ganglioglioma |  | MAP2K1 |  | „tumour flare” |
| infant |  | neuroepithelial |  | Targeted |  | „rapid progression” |
| adolescent |  | xanthoastrocytoma |  | Raf |  | regrowth |
|  |  | neurocytoma |  | Selumetinib |  | cessation |
|  |  |  |  | Trametinib |  | withdraw* |
|  |  |  |  | Binimetinib |  | discontinuat* |
|  |  |  |  | Cobimetinib |  | stop |
|  |  |  |  | mirdametinib |  | taper |
|  |  |  |  | refametinib |  | deescalat* |
|  |  |  |  | Dabrafenib |  |  |
|  |  |  |  | vemurafenib |  |  |
|  |  |  |  | Encorafenib |  |  |
|  |  |  |  | tovorafenib |  |  |
|  |  |  |  | DAY101 |  |  |

+ additional search: first 3 columns only and filters „trial” and “clinial trial” – to search manually information in trials

4. Clinicaltrials.gov

| glioma | AND | Selumetinib | AND | Age: Child |
| --- | --- | --- | --- | --- |
| Low-grade glioma |  | Trametinib |  |  |
| Low grade glioma of brain |  | Binimetinib |  |  |
| Low grade glioma/ LGG |  | Cobimetinib |  |  |
| astrocytoma |  | mirdametinib |  |  |
|  |  | refametinib |  |  |
|  |  | Dabrafenib |  |  |
|  |  | vemurafenib |  |  |
|  |  | Encorafenib |  |  |
|  |  | tovorafenib |  |  |
|  |  | DAY101 |  |  |
|  |  | BRAF |  |  |
|  |  | MAPK |  |  |
|  |  | MAP2K1 |  |  |
|  |  | MEK |  |  |
|  |  | RAF |  |  |

5. ICTRP

| glioma | AND | Selumetinib | AND | Age: Child |
| --- | --- | --- | --- | --- |
| low-grade glioma |  | Trametinib |  |  |
| low grade glioma |  | Binimetinib |  |  |
| astrocytoma |  | Cobimetinib |  |  |
|  |  | mirdametinib |  |  |
|  |  | refametinib |  |  |
|  |  | Dabrafenib |  |  |
|  |  | vemurafenib |  |  |
|  |  | Encorafenib |  |  |
|  |  | tovorafenib |  |  |
|  |  | DAY101 |  |  |
|  |  | BRAF |  |  |
|  |  | MAPK |  |  |
|  |  | MAP2K1 |  |  |
|  |  | MEK |  |  |
|  |  | RAF |  |  |
