# Supplementary material for: Rebound regrowth phenomenon in patients with pediatric low-grade gliomas treated with MAPK inhibitors – a systematic review
Source: Oncol Rev. 2026 Apr 29;20:1815932. doi: 10.3389/or.2026.1815932 (PMC13168116; doi:10.3389/or.2026.1815932)
Supplement: Supplementary file 2 [file Supplementaryfile3.docx]

**Risk of bias assessment – methods**

**Overview**

Risk of bias was assessed for all included studies, including those from which only the individual data of the patients were retrieved. Given the heterogeneity of study designs (early-phase clinical trials, retrospective cohort studies, case series, case reports) and the non-comparative nature of the research question, a single standardized tool was not applicable. Therefore, design-specific Joanna Briggs Institute (JBI) checklists were used as the basis for signaling questions, and results were mapped to predefined bias domains.

**Risk of bias domains**

For each study, five domains of bias were evaluated: (1) selection bias, (2) exposure measurement, (3) outcome measurement, (4) follow-up completeness, and (5) reporting bias. These domains were selected to ensure consistency across heterogeneous study designs and to reflect key sources of bias relevant to observational and descriptive studies.

**Assessment process**

Two reviewers independently assessed each study. For each relevant JBI signaling question, responses were categorized as “Yes”, “No”, “Unclear”, or “Not applicable”. Discrepancies were resolved through discussion and consensus.

**Domain-level risk of bias judgment**

Domain-level risk of bias was determined based on aggregated signaling question responses. A domain was classified as low risk of bias if all responses were “Yes”. Moderate risk of bias was assigned when at least one response was “Unclear” but no items were rated “No”. High risk of bias was assigned when one or more items were rated “No”, indicating substantial methodological limitations.

**Overall risk of bias**

The overall risk of bias for each study was determined using a hierarchical approach. Studies were classified as low risk of bias if all domains were rated as low risk. Moderate risk of bias was assigned if at least one domain was rated as moderate risk and none were high risk. High risk of bias was assigned if at least one domain was rated as high risk.

**Data presentation**

Results of the risk of bias assessment were summarized in tabular form, presenting domain-level and overall judgments for each study.

**Risk of bias assessment – questionnaire template**

**1. Selection Bias**

Were inclusion criteria clearly defined?
[ ] Yes [ ] No [ ] Unclear [ ] Not applicable

Was the sample representative of the population?
[ ] Yes [ ] No [ ] Unclear [ ] Not applicable

Were participants recruited consecutively or completely?
[ ] Yes [ ] No [ ] Unclear [ ] Not applicable

Domain-level judgment: [ ] Low [ ] Moderate [ ] High

**2. Exposure Measurement (MAPKi discontinuation)**

Was MAPKi discontinuation/dose reduction clearly defined?
[ ] Yes [ ] No [ ] Unclear [ ] Not applicable

Was timing of discontinuation specified?
[ ] Yes [ ] No [ ] Unclear [ ] Not applicable

Was exposure measured consistently across participants?
[ ] Yes [ ] No [ ] Unclear [ ] Not applicable

Domain-level judgment: [ ] Low [ ] Moderate [ ] High

**3. Outcome Measurement (Rebound Regrowth)**

Was RR reported according to its definition (per O’Hare et al)?
[ ] Yes [ ] No [ ] Unclear [ ] Not applicable

Were standardized imaging criteria used (e.g., RAPNO/RANO)?
[ ] Yes [ ] No [ ] Unclear [ ] Not applicable

Was outcome assessment applied consistently?
[ ] Yes [ ] No [ ] Unclear [ ] Not applicable

Domain-level judgment: [ ] Low [ ] Moderate [ ] High

**4. Follow-up Completeness**

Was follow-up duration reported?
[ ] Yes [ ] No [ ] Unclear [ ] Not applicable

Was follow-up sufficient to detect RR?
[ ] Yes [ ] No [ ] Unclear [ ] Not applicable

Were losses to follow-up described and acceptable?
[ ] Yes [ ] No [ ] Unclear [ ] Not applicable

Domain-level judgment: [ ] Low [ ] Moderate [ ] High

**5. Reporting Bias**

Were all relevant outcomes reported?
[ ] Yes [ ] No [ ] Unclear [ ] Not applicable

Was the case selection free from apparent publication bias (i.e., not primarily driven by unusual, favorable, or hypothesis-generating outcomes)?
[ ] Yes [ ] No [ ] Unclear [ ] Not applicable

Was individual-level data provided (if applicable)?
[ ] Yes [ ] No [ ] Unclear [ ] Not applicable

Were key clinical variables adequately described?
[ ] Yes [ ] No [ ] Unclear [ ] Not applicable

Domain-level judgment: [ ] Low [ ] Moderate [ ] High

**Overall Risk of Bias**

Overall judgment: [ ] Low [ ] Moderate [ ] High
