# Supplementary material for: Rebound regrowth phenomenon in patients with pediatric low-grade gliomas treated with MAPK inhibitors – a systematic review
Source: Oncol Rev. 2026 Apr 29;20:1815932. doi: 10.3389/or.2026.1815932 (PMC13168116; doi:10.3389/or.2026.1815932)
Supplement: Supplementary file 4 [file Supplementaryfile4.docx]

Risk of Bias Summary Table

| Study  (author, year) | Study design | Selection bias | Exposure measurement | Outcome measurement | Follow-up completeness | Reporting bias | **Overall RoB** |
| --- | --- | --- | --- | --- | --- | --- | --- |
| Aguilera 2016 | Case report | High | Low | High | Low | High | **High** |
| Banerjee 2017 | Prospective Phase I Clinical Trial | Moderate | Low | Moderate | High | Low | **High** |
| Bruggers 2021 | Case series | High | High | High | Low | High | **High** |
| Bruggers 2022 | Retrospective cohort study | High | High | Moderate | High | High | **High** |
| Crentsil 2024 | Case report | High | Low | High | Low | High | **High** |
| del Bufalo 2018 | Case series | Moderate | Low | High | High | Moderate | **High** |
| Hargrave 2019 | Prospective Phase I/IIa Clinical Trial | Moderate | Low | Moderate | Low | Low | **Moderate** |
| Marks 2017 | Case report | High | Low | High | Low | High | **High** |
| Mednansky 2021 | Case report | High | Low | High | Low | High | **High** |
| Nicolaides 2020 | Prospective Phase I Clinical Trial | Moderate | Low | Moderate | Low | Low | **Moderate** |
| Nobre 2020 | Retrospective cohort study | Moderate | Moderate | Moderate | Low | High | **High** |
| Perez 2021 | Retrospective cohort study | Moderate | Low | Low | Moderate | Moderate | **Moderate** |
| Perreault 2024 | Prospective Phase II Clinical Trial | Low | Low | Low | Low | Moderate | **Moderate** |
| Laflamme 2019 | Case series | High | Low | Moderate | Low | High | **High** |
| Ramaglia 2024 | Retrospective cohort study | High | High | Moderate | Low | High | **High** |
| Raswoli 2020 | Case report | High | Low | High | Low | High | **High** |
| Selt 2020 | Retrospective cohort study | High | Low | Moderate | Moderate | Moderate | **High** |
| Toledano 2016 | Case report | High | Low | High | Low | High | **High** |
| Tsai 2022 | Retrospective cohort study | High | Low | Low | Moderate | Low | **High** |
| Warriner 2021 | Retrospective cohort study | High | High | Moderate | Moderate | Low | **High** |
